# School-Level Economic Disparities in Police-Reported Crimes and Active Commuting to School

**DOI:** 10.3390/ijerph182010885

**Published:** 2021-10-16

**Authors:** Katie Burford, Leigh Ann Ganzar, Kevin Lanza, Harold W. Kohl, Deanna M. Hoelscher

**Affiliations:** 1 Michael and Susan Dell Center for Healthy Living, The University of Texas Health Science Center at Houston (UTHealth) School of Public Health in Austin, Austin, TX 78701, USA; Leigh.A.Ganzar@uth.tmc.edu (L.A.G.); Kevin.L.Lanza@uth.tmc.edu (K.L.); Harold.W.Kohl@uth.tmc.edu (H.W.K.III); Deanna.M.Hoelscher@uth.tmc.edu (D.M.H.); 2 Department of Kinesiology and Health Education, The University of Texas at Austin, Austin, TX 78712, USA

**Keywords:** active commuting to school, children, physical activity, disparities, equity, crime, active travel, safety

## Abstract

Perceived safety remains one of the main barriers for children to participate in active commuting to school (ACS). This ecological study examined the associations between the number of police-reported crimes in school neighborhoods and ACS. The percentage of active travel trips was assessed from a teacher tally survey collected from students across 63 elementary schools that were primarily classified as high-poverty (*n* = 27). Geographic Information System (GIS) was used to create a detailed measure of police-reported crimes during 2018 and neighborhood covariates that occurred within a one-mile Euclidean buffer of the schools. Statistical analyses included linear fixed effects regressions and negative binomial regressions. In fully-adjusted models, reported crime did not exhibit significant associations with ACS. Medium-poverty schools were indirectly associated with ACS when compared to high- and low-poverty schools in all models (*p* < 0.05). Connectivity and vehicle ownership were also directly associated with ACS (*p* < 0.05). Low- and medium-poverty schools were indirectly associated with all types of reported crime when compared to high-poverty schools (*p* < 0.05). Although reported crime was not associated with school-level ACS, differences in ACS and reported crime do exist across school poverty levels, suggesting a need to develop and promote safe and equitable ACS interventions.

## 1. Introduction

The 2018 Physical Activity Guidelines for Americans recommends that children participate in 60 min or more of moderate-to-vigorous physical activity daily [1]. However, only an estimated 24% of children met the guidelines in 2016 [2]. To address the inadequate prevalence of physical activity among children, active commuting to school (ACS) represents one environmentally sustainable and accessible opportunity for children to participate in physical activity. Moreover, evidence supports that children are more likely to meet daily physical activity guidelines and have higher levels of daily physical activity when they participate in ACS [3,4,5,6]. Despite the potential of ACS for children’s health, the proportion of children who walked or biked to school in the U.S. fell from 47.7% in 1969 to 10.7% in 2017 [7,8].

To understand the decline in ACS and determine the best strategies for promoting ACS, extensive empirical evidence has used the socio-ecological model (SEM) of health behavior as a framework to measure a range of individual, school, and environmental factors associated with ACS [9]. Crime is one environmental correlate of children’s ACS, which is potentially more modifiable than individual (e.g., sex, race, ethnicity) and school (e.g., distance from home) factors [10,11]. Crime is measured in two ways: (1) self-reported instruments to measure individuals’ perceptions of crime and (2) the spatial and temporal analysis of police-reported crime data. Evidence suggests that these measures should be assessed independently because they each provide a unique variance in their associations with physical activity [12].

The majority of research on crime-related determinants of ACS has centered on parental perceptions of crime, which are consistently indirectly associated with children’s ACS behavior [12,13,14]. However, only two studies with inconsistent findings have examined associations between police-reported crime and ACS [14,15]. Bosch and colleagues (2020) found police-reported crime along the route to school was not significantly associated with children (aged 5–11) participating in ACS [15]. Conversely, Vonderwalde and colleagues (2018) determined that adolescents (aged 10–13) living in neighborhoods with the highest crime rate quartile had a significantly higher prevalence of objectively measured ACS [16]. These data suggest that reported crime incidents may not deter adolescents from participating in ACS, and the authors stated the need to include other confounding variables within their analysis such as household income and vehicle ownership.

One major limitation within the existing literature is the difference in choice of measures for police-reported crime and ACS. Vonderwalde et al. (2019) used accelerometry and Global Positioning System (GPS) loggers to objectively capture ACS [16]. This was also the only study to make a distinction between reported crimes against property (robbery, break and enter, theft, vehicle theft) and against persons (assault and sexual assault) in the measure of crime, which found higher levels of ACS on routes to school with higher quartiles of crime against persons [16]. Reported crime is a complex measure as people are likely to modify their travel behavior in the presence of various types of crime (major vs. minor; property vs. violent; visible vs. not visible) [17]. For example, property crime has been found to deter individuals from placing personal property at risk, while violent crime has significantly altered individual’s experience walking or cycling because they may be exposed to personal threats to safety [17]. Additionally, individuals may be more strongly influenced by crime at their destinations rather than along their routes or in their home neighborhood [17]. As the existing studies focus on exposure to police-reported crime around the home neighborhood and on the route to school, the school neighborhood or the destination of travel for children should also be considered [15,16]. An additional concern in these studies is the relatively small sample size of schools from only two countries: Bosch and colleagues (2020) included 13 schools from London, UK, in their study sample and Vonderwalde and colleagues (2019) studied children across 12 electoral districts in Kingston, Canada [15].

Lastly, the existing literature does not include a measure of school economic disadvantage, which may serve as an important confounding variable in the association of police-reported crime in school neighborhoods and ACS. Only Bosch and colleagues (2017) incorporated a neighborhood-level measure of socio-economic status within their analysis, and showed that neighborhood deprivation (categorized as low, medium, and high deprivation) was not associated with children’s decision to participate in ACS [15]. School-level poverty revealed mixed associations with children participating in ACS [18]. However, Zhu and Lee (2008) did find that school economic disparities exist in reported crime rates surrounding elementary schools in Austin, Texas [19]. Specifically, schools with higher poverty rates also had higher crime rates in school attendance areas, despite the schools being situated in more walkable neighborhoods [19]. These findings underscore that elementary students who attended schools with higher poverty rates may experience unsafe travel conditions when actively commuting. However, the researchers did not capture differences across school poverty levels and used only one spatial scale, which may not be representative of the proximal school neighborhood conditions. Furthermore, the authors’ findings may no longer be representative of the city of Austin’s demographics as the population grew rapidly from 790,390 in 2010 to 961,855 in 2020 [20]. Therefore, this area of research could benefit from an updated and stratified measure of school poverty to understand if there are potential school-level economic disparities related to police-reported crime and children participating in ACS.

Overall, the previous research revealed mixed associations between police-reported crimes in school neighborhoods and children participating in ACS. Thus, the primary purpose of this ecological study was to determine the associations between the number of police-reported crimes in school neighborhoods and children participating in ACS using detailed measures of reported crime and a relatively large sample of diverse schools and neighborhoods. The secondary purpose was to determine the associations between school poverty level and the number of police-reported crimes in school neighborhoods. With this paper, we extend the existing literature by including (1) a large, diverse sample across elementary grades; (2) a measure of different types of police-reported crime in school neighborhoods; and (3) a measure of school poverty level.

## 2. Materials and Methods

### 2.1. Study Setting and Participants

Data were collected during the 2018–2019 school year as part of the Safe TRavel Environment Evaluation in Texas Schools (STREETS) study, which includes both a quasi-experimental cohort study and serial cross-sectional study to evaluate the effects of Safe Routes to School (SRTS) infrastructure projects on student physical activity and the proportion of students that participated in ACS. The STREETS study sample consisted of (*n* = 69) public elementary schools within the Austin, Texas, city limits. Of these, 6 did not have complete secondary data (e.g., police-reported crime, census data). As a result, these were excluded from the analysis and the final sample consisted of 63 schools. Informed consent procedures and study protocol were approved by the institutional review board (IRB) at UTHealth (HSC-SPH-17-0638) and by the research departments at each participating school district. Additionally, all principals provided written consent for schools to participate in STREETS.

### 2.2. Measurement of Active Commuting to School (ACS)

ACS was defined as the percentage of student trips to and from school made by active transport modes (walking or biking) averaged across three consecutive school weekdays. School-level percentage of trips using active transport modes was determined using the standard SRTS student tally method. This method has high reliability (kappa > 0.8) and validity (kappa > 0.75) at all elementary school grades [21]. Over three consecutive school days, teachers asked students about their transport mode to and from school by a show of hands in their classroom. Data were collected from students in third-, fourth-, and fifth-grade classrooms from all participating schools.

### 2.3. Measurement of School Demographics

School demographic information for third- through fifth-grade students (i.e., race/ethnicity and eligibility for free/reduced-price lunch (FRPL) were obtained through data requests from Texas Education Agency [22,23]. The percentage of students eligible for FRPL provided a proxy for the concentration of students from low-income families within a public-school neighborhood catchment area and can be used to determine a school’s relative poverty level. We used the National Center for Education Statistics (NCES) definitions to create a categorical variable for school poverty level [24]. These FRPL categories include low-poverty schools (25.0% or less); mid-low poverty schools (25.1–50.0%); mid-high poverty schools (50.1–75.0%); and high-poverty schools (75.1% or more).

### 2.4. Measurement of School Neighborhood

We defined school neighborhood as a one-mile Euclidean distance around each school. This buffer distance was chosen because evidence suggests that children in the United States who live within one mile of their school were more than three times as likely to participate in ACS compared to children who lived at a greater distance [25]. To understand the choice of spatial scale, we performed a sensitivity analysis using a two-mile buffer around each school because students are eligible for a transportation route service if they live within two miles of their school [26]. We worked within a Geographic Information System (ArcGIS10.8, ESRI, Redlands, CA, USA) to calculate the number of police-reported crimes that occurred in 2018, population density, household income, neighborhood connectivity, and vehicle ownership within a one-mile buffer of each school.

### 2.5. Measurement of Police-Reported Crime

Data for the number of police-reported crimes within a one-mile buffer of each school that occurred in 2018 throughout Austin originated from the City of Austin [27]. We used the FBI Uniform reporting definitions to develop five independent variables for police-reported crime occurring in 2018 within a one-mile buffer of each school: total, Part I (minor), Part II (major), property, and violent [28]. Minor crimes include incidents such as drugs, simple assaults, and public intoxication. Major crimes represent a sum of property and violent crimes. Property crimes include burglary, theft, and auto theft. Violent crimes include murder, rape, robbery, and aggravated assault.

### 2.6. Measurement of Population Density, Household Income, and Neighborhood Connectivity

Population density, household income, neighborhood connectivity, and vehicle ownership were obtained from 2018 U.S. Census Bureau five-year block group estimates [29]. Population density was defined as the total population count within a one-mile buffer of each school. Household income was defined in U.S. dollars as the median household income within a one-mile buffer of each school. Neighborhood connectivity was defined as a count of three- and four-way intersections using a road network within a one-mile buffer of each school. Vehicle ownership was defined as the percentage of households with one or more vehicles within a one-mile buffer of each school. We identified these potential covariates from the literature as having significant associations with ACS [30,31,32].

### 2.7. Statistical Analysis

We performed descriptive statistics to determine frequencies with proportions, means with standard deviations (±SD), and medians with interquartile range (IQR). All variables were summarized at the school-level. For the primary aim, we used five generalized linear fixed models to analyze the associations between the number of police-reported crimes (total, minor, major, property, violent) and ACS adjusting for neighborhood-level covariates and clustering within schools. Prior to estimating final models, we first determined crude associations for neighborhood- and school-level characteristics by including each variable in a separate model. Due to issues of multicollinearity (variance inflation factor > 5), we excluded neighborhood household income from the final models. Intraclass correlation coefficients (ICCs) revealed the clustering of ACS within schools. For the secondary aim, we used negative binomial regression modeling to predict the associations between the number of police-reported crimes and school poverty level to account for the overdispersion of police-reported crime variables. To aid in interpretability, we reported negative binomial regression model results as incidence-rate ratios (i.e., exponentiated standardized regression coefficients). For all regression analyses, we standardized all independent variables, thus associations were reported as standardized regression coefficients (β) and 95% confidence intervals (95% CI). We performed a sensitivity analysis using a two-mile buffer around each school because students are eligible for a transportation route service if they live within two miles of their school [26]. A two-sided *p*-value < 0.05 was considered statistically significant. We conducted all statistical analyses using R studio Version 1.4.1717 and R version 4.10 in 2021.

## 3. Results

### 3.1. School- and Neighborhood-Level Descriptive Characteristics

We collapsed the mid-low and mid-high poverty level schools to a medium-poverty level (25.1–75.0%) as there were no differences in results of the analytical analyses. Most of the schools in the analytic sample (*n* = 63) were classified as high-poverty-level schools (*n* = 27) when compared to low-poverty- (*n* = 12) and medium-poverty- (*n* = 22) level schools. Students from the schools were primarily Hispanic/Latino (84.3%) and a median of 12.21% of trips were made by active travel modes (Table 1).

The median number of total police-reported crimes in school neighborhoods was 1564, with most of these crimes reported as minor crimes (median = 1016) as compared to major crimes (median = 522). More property crimes (median = 426) were reported than violent crimes (median = 40). The median population density in school neighborhoods was 2227.1 persons; the median household income was $64,730.80; the median percentage of households with one or more cars was 94.1%, and on average there were 260 three- and four-way intersections. Low-poverty schools had a higher percentage of households that owned a car (median = 98.1) compared to medium- (median = 95.5) and high- (median = 91.8) poverty-level schools. However, medium-poverty-level schools had higher neighborhood connectivity (mean = 270.2, SD = 131.5) compared to low- (mean = 248, SD = 126.6) and high- (mean = 258, SD = 75.2) poverty-level schools.

### 3.2. Associations with ACS

**Figure 1:**
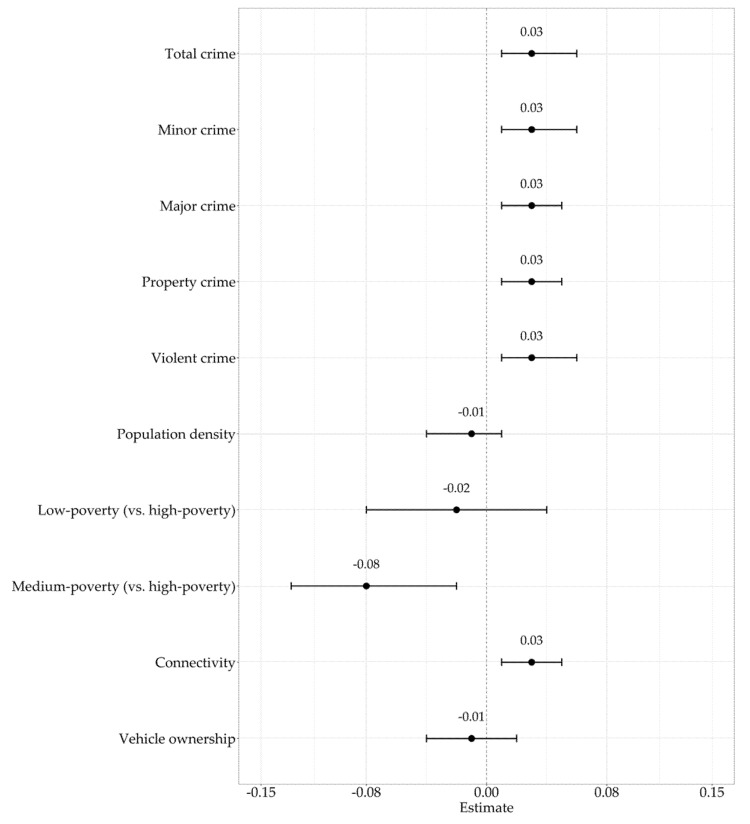
Unadjusted models for police-reported crime types and ACS.

In unadjusted models (Figure 1, Table A1), total crime (β = 0.03, 95% CI: 0.01–0.06), minor crime (β = 0.03, 95% CI: 0.01–0.06), major crime (β = 0.03, 95% CI: 0.01–0.05), property crime (β = 0.03, 95% CI: 0.01–0.05), and violent crime (β = 0.03, 95% CI: 0.01–0.06) were all significant and directly associated with the percentage of children ACS. Medium-poverty-level schools (β = −0.08, 95% CI: −0.13–0.02) were significant and indirectly associated with the percentage of children ACS when compared to high-poverty-level schools. Connectivity (β = 0.03, 95% CI: 0.01–0.05) was significant and directly associated with the percentage of children ACS. ICC for percent ACS for schools was 0.48 in all models.

### 3.3. Associations with ACS

**Figure 2:**
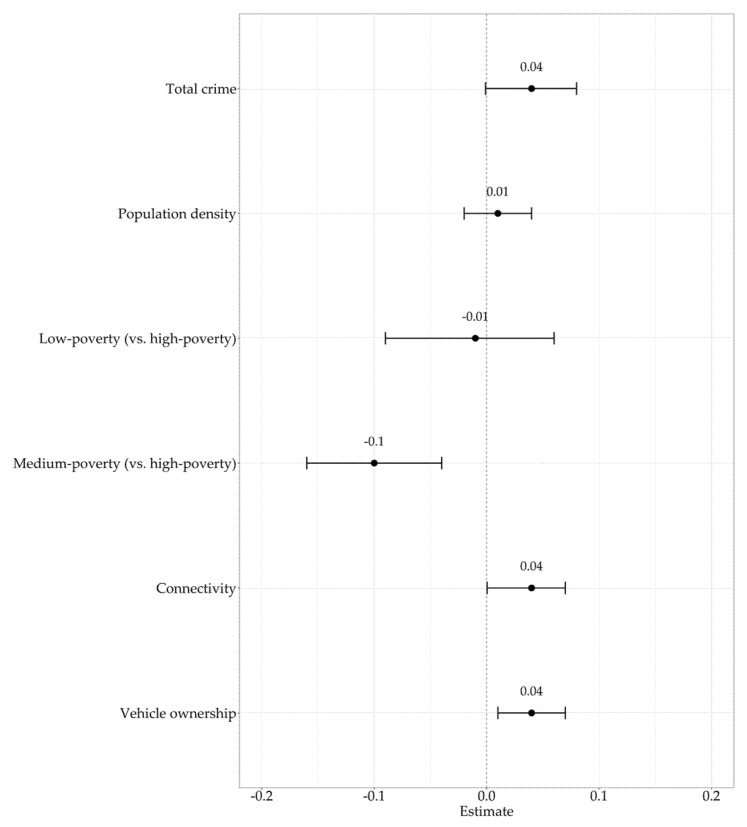
Mutually adjusted model for total police-reported crime and ACS.

In fully adjusted models (Table A2), results revealed that total crime (β = 0.04; 95% CI: −0.0001–0.08; *p* = 0.06; Figure 2), minor crime (β = 0.04; 95% CI: −0.0001–0.07; *p* = 0.06), major crime (β = 0.03; 95% CI: −0.01–0.07; *p* = 0.10), property crime (β = 0.03; 95% CI: −0.01–0.08; *p* = 0.10), and violent crime (β = 0.02, 95% CI: −0.01–0.06, *p* = 0.16) were not significantly associated with ACS. Medium-poverty-level schools were significant and indirectly associated with ACS compared to low-poverty schools and high-poverty schools in all models (*p* = 0.00). There were no significant differences in the associations between ACS and high- and low-poverty schools (all *p* > 0.05). Connectivity and vehicle ownership were also significant and directly associated with ACS in all models (*p* < 0.05), except connectivity was not significantly associated with ACS when adjusted for property crime (*p* = 0.06). There were no significant interactions between all types of reported crime and other covariates (*p* > 0.05). The medium-poverty-level standardized coefficient had the greatest magnitude, which suggests it is the most significant predictor of ACS for all models [33].

The sensitivity analysis, using a two-mile Euclidean buffer to capture the school neighborhood, showed that in all models only medium-poverty-level exhibited a significant and indirect association with ACS when compared to low-poverty schools and adjusting for covariates (*p* < 0.05).

### 3.4. Associations with Police-Reported Crime

In adjusted negative binomial regression models (Table 2), both low-poverty and medium-poverty schools were significant and indirectly associated with total minor, major, property, and violent crimes compared to high-poverty schools (*p* < 0.05). In other words, the incident rates of total, minor, major, property, and violent crimes were 76%, 79%, 68%, 64%, and 90% lower, respectively, for low-poverty schools compared to high-poverty schools. Additionally, the incident rates of total, minor, major, property, and violent crimes were 32%, 47%, 20%, 14%, and 51% lower, respectively, for medium-poverty schools compared to high-poverty schools.

## 4. Discussion

This study examined the ecological associations between different types of police-reported crime and active commuting to school of elementary-aged children at the school-level. We found reported crimes of any type were not significantly associated with ACS in the adjusted models. Bosch and colleagues (2020) similarly found no significant associations between police-reported crime and active commuting among elementary school children after adjusting for built environment and socio-demographic characteristics [15]. Therefore, our results support the conclusion that there is no association between police-reported crime and elementary children participating in ACS when adjusted for other school- and neighborhood-level factors.

However, there were school economic differences related to ACS. Children from medium-poverty schools were less likely to participate in ACS compared to high- and low-poverty schools. It is consistently hypothesized that rates of walking to school are highest among schools with a greater percentage of children enrolled in the Free and Reduced Price Meal program [34]. Similarly, Bosch and colleagues (2017) found that children from affluent families were less likely to engage in ACS compared to less affluent families but there were no differences between children from high-, medium-, and low-deprivation neighborhoods [15]. Molina-Garcia and Queralt (2017) also showed that children attending schools in lower SES neighborhoods reported more ACS trips per week than those attending higher SES neighborhoods [35]. These findings might be explained by children from high-poverty schools not having a choice when commuting to school as low-income families are less likely to own a vehicle and are more likely to be single-parent households [18]. In contrast, the finding that children from low-poverty-level schools were also more likely to actively commute to school compared to medium-poverty-level schools is less commonly supported in the literature [18]. We speculate that children from low-poverty (e.g., higher income) schools may have reported more active travel modes in this sample because they may have increased access to opportunities that facilitate ACS (e.g., bike ownership, sidewalks, green-space) or parents who have more positive perceptions of ACS. Other social environment factors that may vary across neighborhoods, such as social capital and collective efficacy, could provide additional insights into these inferences and should be explored in subsequent studies. Lastly, future findings from the STREETS study may help to explain these economic differences in ACS.

The results of the secondary purpose revealed that school-level economic disparities are associated with different types of police-reported crimes that occurred in elementary school neighborhoods. Unsurprisingly, there was a greater number of all types (total, minor, major, property, violent) of reported crimes in school neighborhoods of high- and medium-poverty-level schools compared to low-poverty-level schools. High-poverty-level school neighborhoods showed the highest number of all reported crime types. This corresponds to the existing literature in that low-income neighborhoods suffer disproportionately higher rates of crime and violence [36]. Likewise our results matched Zhu and Lee’s (2008) findings that Austin elementary schools with higher poverty rates had higher crime rates in attendance areas, but we extended these findings by showing that differences exist across school poverty levels and within one-mile of each school [19]. Children from low-income neighborhoods are also significantly more likely to witness severe violence than youths from middle- and high-income neighborhoods, which is supported by our findings of police-reported violence being highest around high-poverty schools [36]. As we also found that children from high-poverty schools were more likely to participate in ACS compared to children from medium-poverty schools, there may also be more opportunities for exposure to crime and violence along commutes [37]. This is a public health concern because exposure to violence puts youths at risk of experiencing physical harm, long-term mental illness, and delayed development [38]. Therefore, SRTS strategies (e.g., walking school buses, corner captains, safe havens, safe passages) and other evidence-based initiatives (e.g., mentorship programs, crime prevention through environmental design), which prevent crime and violence and improve safety, should be strongly considered in these low- and middle-income neighborhoods surrounding schools [39].

In contrast to the null finding between police-reported crime and ACS, parental perception of crime is consistently inversely associated with ACS [12,13,14,15]. The inconsistency in results between perception versus objectively measured crime may stem from existing heterogeneity across methods of measurement for these exposures and active travel outcomes [13]. In fact, a recent systematic review determined that the existing studies measuring crime and children’s active mobility behavior are moderate or weak in quality due to methodological differences, which may impact the reliability of evidence [13]. To improve this area of research, Zougheibe et al. (2021) suggested that questionnaires measuring perception of safety be redesigned to (1) include a temporal and geographical component; (2) capture frequency and intensity of fear related to crime; (3) assign a timeframe to questions [13]. Additionally, the standardization of protocols using accelerometers, GPS, and spatial-temporal data may improve our evidence of measured crime and ACS [13]. As there are a limited number of studies exploring both perceived and actual crime and their associations with ACS, this is an area ripe for future research. It may be that actual crime influences children’s ACS directly and indirectly through perception of crime.

This study is not without limitations. As we sought to explore a large sample of school- and neighborhood-level associations with aggregated active commuting to school data, temporality and causality were not established. To mitigate potential temporal bias, future studies could ensure that police-reported crime proceeded active travel trips by including additional proceeding years of crime data. Furthermore, ecological fallacy is a potential limitation of the associations presented in this study. However, the use of ecological analyses was appropriate for determining the need for active commuting to school and crime-prevention interventions across schools. Future studies could validate these findings by using multilevel analyses to examine the joint role of individual and aggregate exposure to reported crime. Second, self-reported ACS data could not be confirmed objectively but there is still no consensus on the best measure of ACS [18]. Additionally, there are limitations to existing measures of police-reported crime, which tend to be underestimated and may not reflect an individual’s lived experience [40]. We did not include other individual and environmental predictors of ACS such as distance and perception of crime, which may more thoroughly explain these associations and lead to residual confounding in these associations [18]. Finally, these findings may only generalize to other samples of schools with similar demographic and environmental compositions as those in our study in Austin, Texas, which will be important to explore in future research.

## 5. Conclusions

We explored the ecological associations between the number of police-reported crimes in school neighborhoods and children’s ACS from a large sample of diverse schools in Austin, Texas. At the school-level, we found that all types of police-reported crime were not associated with ACS in adjusted models, but school poverty level was an important determinant of children who participated in ACS. Children attending medium-poverty schools reported less ACS than those attending low- and high-poverty schools. High- and medium-poverty schools had a significantly higher number of all types of police reported-crimes (total, minor, major, property, violent), which may expose children from lower economic status schools to greater violence or criminal acts on their commutes to school and lead to other long-term health consequences. The ecological design allowed for studying an important population subgroup—disadvantaged and advantaged school neighborhoods across a large U.S. metropolitan city—that needs to be considered in future efforts to monitor ACS and safety outcomes and in designing ACS interventions to eliminate health inequities. Further, as safety and equity are integral components of SRTS initiatives, this study builds the evidence base for policymakers, planners, and engineers to address these school-level economic disparities in ACS and reported crime and the overall low proportion of children ACS. More studies considering disparities related to children’s safety while ACS are also needed to improve physical activity and safety outcomes.

## Figures and Tables

**Table 1:** School- and Neighborhood- Level Descriptive Characteristics by School Poverty Level.

	Low-Poverty (*n* = 14)	Medium-Poverty (*n* = 22)	High-Poverty (*n* = 27)	Total (*n* = 63)
**School Characteristics**				
Race/Ethnicity (%, Median (IQR))				
White	93 (22.1)	12.2 (25.6)	3.5 (3)	9.6 (46.9)
Black	1.2 (1.1)	3.5 (2.6)	2.8 (1.0)	2.8 (1.4)
Hispanic/Latino	5.2 (21.6)	69.1 (26.0)	93.4 (4.2)	84.3 (55.4)
Trips to/from school by active travel modes (%, Median (IQR))	16.0 (8.6)	6.1 (9.5)	14.7 (14.3)	12.21 (14.6)
**Neighborhood Characteristics**				
Total Crime (Median (IQR))	434 (834.2)	1375 (1468)	2325 (1452.5)	1564 (1872.5)
Minor Crime (Median (IQR))	191 (471.3)	920.5 (824.7)	1662 (996)	1016 (1361)
Major Crime (Median (IQR))	197.5 (346.2)	464 (513.8)	737 (591.5)	522 (704)
Property Crime (Median (IQR))	177.5 (349.8)	429.5 (433)	612 (539.5)	426 (602.5)
Violent Crime (Median (IQR))	7.5 (14.8)	32 (45)	77 (92.5)	40 (66.5)
Population Density (Median (IQR))	2241.3 (2808.5)	2281.1 (1667.0)	2203.8 (742.6)	2227.1 (1230.5)
Household Income ($, Median (IQR))	121,036.4 (169,044.5)	77,780.87 (124,473.1)	52,108.8 (11,452.1)	64,720.8 (25,027.6)
Vehicle Ownership (%, Median (IQR))	98.1 (4.3)	95.5 (5.1)	91.8 (4.0)	94.1 (6.4)
Connectivity (*n*, Mean (SD))	248 (126.6)	270.2 (131.5)	258 (75.2)	260 (108)

Note: IQR: interquartile range; SD: standard deviation.

**Table 2:** Mutually adjusted models for police-reported crime types and school poverty.

	Dependent Variable:				
	Total Crime	Minor Crime	Major Crime	Property Crime	Violent Crime
Low-poverty (ref = high-poverty)	0.24 *	0.20 *	0.32 *	0.36 *	0.10 *
	(0.21, 0.27)	(0.18, 0.23)	(0.29, 0.37)	(0.32, 0.41)	(0.09, 0.12)
Medium-poverty (ref = high-poverty)	0.68 *	0.62 *	0.80 *	0.86 *	0.49 *
	(0.60, 0.76)	(0.55, 0.70)	(0.71, 0.91)	(0.76, 0.98)	(0.42, 0.57)
Population density	0.52 *	0.53 *	0.49*	0.48 *	0.51 *
	(0.49, 0.54)	(0.51, 0.56)	(0.46, 0.51)	(0.46, 0.98)	(0.48, 0.55)
Intercept	7.54 *	7.19 *	6.33 *	6.14 *	4.33 *
	(7.46, 7.62)	(7.11, 7.27)	(6.24, 6.41)	(6.05, 6.23)	(4.23, 4.43)

Note: Exponentiated standardized regression coefficients (β); 95% CI in parentheses, * *p* < 0.05.

## Data Availability

City of Austin crime report 2018 data available online at https://data.austintexas.gov/Public-Safety/Crime-Reports-2018/vmn9-3bvu (accessed on 20 May 2021); United States Census 2014–2018 American Community Survey 5-Year Estimates available online at https://data.census.gov/cedsci/table?d=ACS%205-Year%20Estimates%20Data%20Profiles&table=DP05&tid=ACSDP5Y2018.DP05&g=0400000US22 (accessed on 31 May 2021).

## Appendix A

**Table A1:** Unadjusted models for police-reported crime types and ACS.

	Dependent Variable:								
	Active Commuting to School (%)								
	(1)	(2)	(3)	(4)	(5)	(6)	(7)	(8)	(9)
Total crime	0.03 *								
	(0.01, 0.06)								
Minor crime		0.03 *							
		(0.01, 0.06)							
Major crime			0.03 *						
			(0.01, 0.05)						
Property crime				0.03 *					
				(0.01, 0.05)					
Violent crime					0.03 *				
					(0.01, 0.06)				
Population density						−0.01			
						(−0.04, 0.01)			
Low-poverty (ref = high-poverty)							−0.02		
							(−0.08, 0.04)		
Medium-poverty (ref = high-poverty)							−0.08 *		
							(−0.13, −0.02)		
Connectivity								0.03 *	
								(0.01, 0.05)	
Vehicle ownership									−0.01
									(−0.04, 0.02)
Intercept	0.13 *	0.13 *	0.13 *	0.13 *	0.13 *	0.14 *	0.17 *	0.13 *	0.14 *
	(0.11, 0.15)	(0.11, 0.15)	(0.11, 0.15)	(0.11, 0.15)	(0.11, 0.16)	(0.11, 0.16)	(0.13, 0.20)	(0.11, 0.16)	(0.11, 0.16)

Note: reported standardized regression coefficients (β); 95% CI in parentheses; * *p* < 0.05.

**Table A2:** Mutually adjusted models for police-reported crime types and ACS.

	Dependent Variable:				
	Active Commuting to School (%)				
	(1)	(2)	(3)	(4)	(5)
Total crime	0.04				
	(−0.001, 0.08)				
Minor crime		0.04			
		(−0.001, 0.07)			
Major crime			0.03		
			(−0.01, 0.07)		
Property crime				0.03	
				(−0.01, 0.08)	
Violent crime					0.02
					(−0.01, 0.06)
Population density	0.01	0.01	0.01	0.01	0.01
	(−0.02, 0.04)	(−0.02, 0.04)	(−0.01, 0.04)	(−0.01, 0.04)	(−0.02, 0.04)
Low-poverty (ref = high-poverty)	−0.01	−0.01	−0.03	−0.03	−0.02
	(−0.09, 0.06)	(−0.09, 0.07)	(−0.10, 0.04)	(−0.10, 0.04)	(−0.10, 0.05)
Medium-poverty (ref = high-poverty)	−0.10 *	−0.10 *	−0.11 *	−0.11 *	−0.10 *
	(−0.16, −0.04)	(−0.16, −0.04)	(−0.17, −0.05)	(−0.17, −0.06)	(−0.16, −0.04)
Connectivity	0.04 *	0.04 *	0.04 *	0.04	0.05 *
	(0.0004, 0.07)	(0.003, 0.07)	(0.001, 0.07)	(−0.001, 0.07)	(0.01, 0.08)
Vehicle ownership	0.04 *	0.04 *	0.04 *	0.04 *	0.04 *
	(0.01, 0.07)	(0.01, 0.07)	(0.01, 0.07)	(0.01, 0.08)	(0.01, 0.07)
Intercept	0.17 *	0.17 *	0.18 *	0.18 *	0.17 *
	(0.13, 0.21)	(0.13, 0.21)	(0.14, 0.22)	(0.14, 0.22)	(0.13, 0.22)

Note: reported standardized regression coefficients (β); 95% CI in parentheses; * *p* < 0.05.

## References

[1] Piercy K.L., Troiano R.P., Ballard R.M., Carlson S.A., Fulton J.E., Galuska D.A., George S.M., Olson R.D. (2018). The Physical Activity Guidelines for Americans. JAMA.

[2] Data Resource Center for Child and Adolescent Health (2016). 2016 National Survey of Childrens Health Data.

[3] Alexander L.M., Inchley J., Todd J., Currie D., Cooper A.R., Currie C. (2005). The Broader Impact of Walking to School among Adolescents: Seven Day Accelerometry Based Study. BMJ.

[4] Cooper A.R., Andersen L.B., Wedderkopp N., Page A.S., Froberg K. (2005). Physical Activity Levels of Children Who Walk, Cycle, or Are Driven to School. Am. J. Prev. Med..

[5] Rosenberg D.E., Sallis J.F., Conway T.L., Cain K.L., McKenzie T.L. (2006). Active Transportation to School over 2 Years in Relation to Weight Status and Physical Activity. Obesity.

[6] Southward E.F., Page A.S., Wheeler B.W., Cooper A.R. (2012). Contribution of the School Journey to Daily Physical Activity in Children Aged 11–12 Years. Am. J. Prev. Med..

[7] McDonald N.C., Brown A.L., Marchetti L.M., Pedroso M.S. (2011). US school travel, 2009: An assessment of trends. Am. J. Prev. Med..

[8] Kontou E., McDonald N.C., Brookshire K., Pullen-Seufert N.C., LaJeunesse S.U.S. (2020). Active School Travel in 2017: Prevalence and Correlates. Prev. Med. Rep..

[9] James S., Owen N. (2002). Ecological models of health behavior. Health Behavior and Health Education: Theory, Research and Practice.

[10] Davison K.K., Werder J.L., Lawson C.T. (2008). Children’s Active Commuting to School: Current Knowledge and Future Directions. Prev. Chronic. Dis..

[11] Lee C., Moudon A.V. (2004). Physical Activity and Environment Research in the Health Field: Implications for Urban and Transportation Planning Practice and Research. J. Plan. Lit..

[12] Orstad S.L., McDonough M.H., Stapleton S., Altincekic C., Troped P.J. (2017). A Systematic Review of Agreement between Perceived and Objective Neighborhood Environment Measures and Associations with Physical Activity Outcomes. Environ. Behav..

[13] Zougheibe R., Xia J., Dewan A., Gudes O., Norman R. (2021). Children’s Outdoor Active Mobility Behaviour and Neighbourhood Safety: A Systematic Review in Measurement Methods and Future Research Directions. Int. J. Health Geogr..

[14] Kerr J., Rosenberg D., Sallis J.F., Saelens B.E., Frank L.D., Conway T.L. (2006). Active Commuting to School: Associations with Environment and Parental Concerns. Med. Sci. Sports Exerc..

[15] Bosch L.S.M.M., Wells J.C.K., Lum S., Reid A.M. (2020). Associations of the Objective Built Environment along the Route to School with Children’s Modes of Commuting: A Multilevel Modelling Analysis (the SLIC Study). PLoS ONE.

[16] Vonderwalde M., Cox J., Williams G.C., Borghese M.M., Janssen I. (2019). Objectively Measured Crime and Active Transportation among 10–13 Year Olds. Prev. Med. Rep..

[17] Appleyard B.S., Ferrell C.E. (2017). The Influence of Crime on Active & Sustainable Travel: New Geo-Statistical Methods and Theories for Understanding Crime and Mode Choice. J. Transp. Health.

[18] Rothman L., Macpherson A.K., Ross T., Buliung R.N. (2018). The Decline in Active School Transportation (AST): A Systematic Review of the Factors Related to AST and Changes in School Transport over Time in North America. Prev. Med..

[19] Zhu X., Lee C. (2008). Walkability and Safety around Elementary Schools. Am. J. Prev. Med..

[20] U.S. Census Bureau (2019). Quick Facts Austin City, Texas.

[21] McDonald N.C., Dwelley A.E., Combs T.S., Evenson K.R., Winters R.H. (2011). Reliability and Validity of the Safe Routes to School Parent and Student Surveys. Int. J. Behav. Nutr. Phys. Act..

[22] Texas Education Agency (2019). National School Lunch Program Eligiblity Data.

[23] Texas Education Agency (2019). Student Enrollment Reports.

[24] U.S. Department of Education, National Center for Education Statistics (2020). Concentration of Public School Students Eligible for Free or Reduced-Price Lunch.

[25] McMillan T.E. (2007). The Relative Influence of Urban Form on a Child’s Travel Mode to School. Transp. Res. Part Policy Pract..

[26] Transportation Policy. https://www.austinisd.org/transportation/policy.

[27] (2018). Crime Reports. https://data.austintexas.gov/Public-Safety/Crime-Reports-2018/vmn9-3bvu.

[28] Austin Police Department (2019). Annual Crime and Traffic Report: 2018 Preliminary Report.

[29] United States Census Bureau Explore Census Data 2014–2018 American Community Survey 5-Year Estimates. Table DP05 ACS Demographic and Housing Estimates. https://data.census.gov/cedsci/table?d=ACS%205-Year%20Estimates%20Data%20Profiles&table=DP05&tid=ACSDP5Y2018.DP05&g=0400000US22.

[30] Wong B.Y.-M., Faulkner G., Buliung R. (2011). GIS Measured Environmental Correlates of Active School Transport: A Systematic Review of 14 Studies. Int. J. Behav. Nutr. Phys. Act..

[31] Mcdonald N. (2007). Active Transportation to School Trends among U.S. Schoolchildren, 1969–2001. Am. J. Prev. Med..

[32] Pont K., Ziviani J., Wadley D., Bennett S., Abbott R. (2009). Environmental Correlates of Children’s Active Transportation: A Systematic Literature Review. Health Place.

[33] Siegel A. (2016). Multiple Regression. Practical Business Statistics.

[34] Su J.G., Jerrett M., McConnell R., Berhane K., Dunton G., Shankardass K., Reynolds K., Chang R., Wolch J. (2013). Factors Influencing Whether Children Walk to School. Health Place.

[35] Molina-García J., Queralt A. (2017). Neighborhood Built Environment and Socioeconomic Status in Relation to Active Commuting to School in Children. J. Phys. Act. Health.

[36] Brownson R.C., Baker E.A., Housemann R.A., Brennan L.K., Bacak S.J. (2001). Environmental and Policy Determinants of Physical Activity in the United States. Am. J. Public Health.

[37] McDonald N.C. (2008). Critical Factors for Active Transportation to School Among Low-Income and Minority Students. Am. J. Prev. Med..

[38] Mazza J.J., Reynolds W.M. (1999). Exposure to Violence in Young Inner-City Adolescents: Relationships with Suicidal Ideation, Depression, and PTSD Symptomatology. J. Abnorm. Child Psychol..

[39] Liberman M., Zimmerman S. (2015). Taking Back the Streets and Sidewalks.

[40] Myers S. (1980). Why Are Crimes Underreported? What Is the Crime Rate? Does It Really Matter?. Univ. Tex. Press.

